# Treatment of painful calciphylaxis with methadone in a palliative care unit: A case report 

**DOI:** 10.5414/CNCS110985

**Published:** 2023-01-12

**Authors:** Shannon Jade King, Jayamangala Sampath Kondasinghe

**Affiliations:** 1 Bethesda Hospital, Claremont, and; 2 Sir Charles Gairdner Hospital, Nedlands, WA, Australia

**Keywords:** calciphylaxis, hemodialysis, pain, methadone, palliative care

## Abstract

A 72-year-old female was admitted with severe calciphylaxis-associated bilateral leg pain on a background of end-stage renal failure on hemodialysis. Palliative care input was requested, and following transfer to our unit she was commenced on low-dose methadone as adjunctive analgesic therapy. A remarkable and sustained analgesic response was observed. Calciphylaxis is associated with severe pain, and careful consideration of analgesic agents and their pharmacokinetics in patients with end-stage renal failure is required.

## Background 

Calcemic uremic arteriolopathy, commonly referred to as calciphylaxis, is a relatively rare and poorly understood condition that portends high morbidity and mortality. It is most commonly observed in patients with renal disease who are receiving dialysis, and poses significant challenges from a pain and wound management perspective [1]. Owing to the rare and often fatal nature of the condition, there is a lack of evidence from high-quality trials to guide management. 

## Case report 

A 72-year-old Caucasian female was admitted to our Palliative Care Unit (PCU) for ongoing management of severe calciphylaxis-associated bilateral leg pain. She had a history of suboptimally controlled type 2 diabetes mellitus (T2DM) in the context of metabolic syndrome and morbid obesity. Concurrent hypertension, dyslipidemia, non-alcoholic fatty liver disease, heart failure with reduced ejection fraction, and smoking-related chronic obstructive pulmonary disease were present. She was taking warfarin for atrial fibrillation. As a result of symptomatic end-stage renal failure (ESRF) related to diabetic and hypertensive nephropathy, she was commenced on hemodialysis 4 years prior. She had ESRF-related anemia and secondary hyperparathyroidism, and had been frequently hospitalized over the previous 3 years with acute issues related to ESRF and diabetes. 

In January 2021, she was admitted to a tertiary hospital for 5 months with a painful left anterior lower leg wound. Initially diagnosed as polymicrobial necrotizing fasciitis, she went to the operating room for debridement of the wound 3 times and required prolonged intensive care unit admissions and intravenous antibiotic therapy. Histopathological results confirmed the presence of calciphylaxis. Her major left leg wound was closed with a full thickness skin graft, and multiple other bilateral leg wounds required frequent dressings and extensive plastic surgery and wound specialist input. Her warfarin therapy was changed to low-dose apixaban, and she commenced intravenous sodium thiosulfate post hemodialysis sessions. 

She was readmitted within a month following discharge with bilateral lower limb pain. Involvement of the Acute Pain Service was employed, and she was subsequently discharged following modifications to her analgesic regimen, only to be again readmitted 4 days later following a fall occurring in the setting of severe pain. She was subsequently referred to the palliative care team at her local hospital for review prior to being transferred to our PCU. 

**Figure 1.**
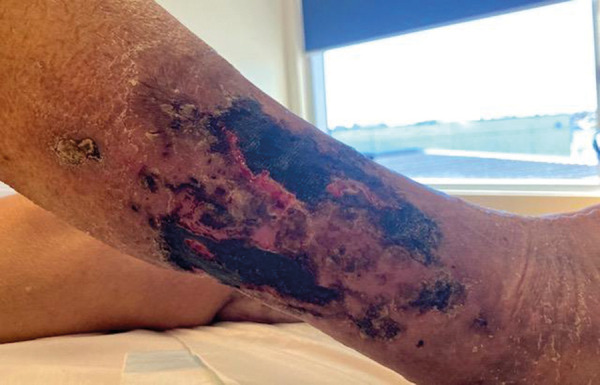
Patient’s distal right anterolateral leg.

On arrival to our PCU, she displayed signs of opioid-induced neurotoxicity with drowsiness, confusion, and myoclonic jerks. Review of clinical notes from her referring hospital indicated that she had received escalating doses of tapentadol sustained release (SR) up to 100 mg twice daily during the 2 weeks prior, which had then been changed to oxycodone SR 20 mg twice daily 4 days prior, and further changed to a fentanyl 12 µg/hour patch the day prior to transfer. In addition, she had been receiving pro re nata (PRN) oxycodone immediate release (IR) 5 mg 4-hourly until the day prior to admission, when her PRN regimen was changed to sublingual fentanyl 100 µg 2-hourly in addition to oxycodone IR 5 mg 4-hourly. She was also receiving pregabalin 75 mg thrice weekly post hemodialysis. Her altered mental status precluded a complete pain history on admission, though she was clearly in distress with any movement or light touch (including of an overlying bed sheet) of her distal lower limbs. Examination of her lower limbs revealed bilateral, widespread patchy areas of superficial ulceration and necrotic eschar formation without evidence of active infection (Figure 1). Diligent wound care, as per the plan from the wound care specialist from her tertiary hospital, was prioritized throughout her admission. 

Initially, her fentanyl patch, pregabalin, and breakthrough sublingual fentanyl were continued at the same doses. As her confusion and drowsiness improved over the following 72 hours, a more complete pain history was obtained, indicating mixed nociceptive and neuropathic pain. She described a constant, distressing ache to both distal legs (worse on the right vs. left; scored at 5 – 6/10 on a visual analogue scale (VAS)), with predictable sharp incident pain associated with any movement of the legs or pressure applied to the regions of her calciphylaxis wounds (scored at 8 – 10/10 on VAS), as well as unprovoked exacerbations of breakthrough pain. In addition, she described burning discomfort variably to both feet and shins. 

Despite cautious, sequential increasing doses of her fentanyl patch to 25 µg/hour, breakthrough PRN sublingual fentanyl to 200 µg and pregabalin to 25 mg twice daily on non-hemodialysis days; and 25 mg in the morning plus 75 mg at night on days of hemodialysis; no major improvements were appreciated in her pain. Two weeks following admission to our unit, we initiated adjunctive therapy with oral methadone at a dose of 2.5 mg daily, noting her baseline QTc was 446 ms. Regular and PRN analgesia were continued at the same doses following initiation of methadone. A remarkable and sustained improvement in her pain was observed following commencement of methadone; after 3 days, her background pain had reduced to 2/10, after 1 week her pain had virtually disappeared to 0 – 1/10 on VAS with no breakthrough PRN analgesic requirements. 

Following resolution of her opioid-induced neurotoxicity there was no evidence of sustained cognitive impairment. Careful discussions were held regarding her multiple life-limiting illnesses and her expected prognosis (estimated to be months, possibly up to a year). She was clear that she still valued her present quality of life and did not wish to cease hemodialysis. Following a 1-month stay in our unit, she was discharged to a residential aged care facility. She was referred to a community palliative care service for ongoing follow-up. 

## Discussion 

### Calciphylaxis 

Calciphylaxis is rare and life-threatening condition, with a mortality rate as high as 60 – 80% [2]. As in the case of our patient, it is usually observed in patients with renal disease and secondary hyperparathyroidism, though can occur in patients without renal or parathyroid disease [2]. Our patient had multiple other risk factors for development of calciphylaxis including female gender, obesity, diabetes mellitus, dialysis dependency, and warfarin use [2]. The pathophysiological mechanisms are poorly understood; however, the condition is characterized histologically by calcification of cutaneous vasculature [2]. A violaceous rash precedes ischemic cutaneous ulcers, which are typically extremely painful [1]. 

Mortality is related to wound infection, sepsis, and organ failure [2]. Aside from wound care, management of calciphylaxis includes treatment of calcium, phosphate, and parathyroid hormone abnormalities, discontinuation of medications associated with calciphylaxis (including warfarin), and intravenous sodium thiosulfate [2]. 

### Pain management in ESRF and calciphylaxis 

Calciphylaxis-associated pain results from tissue damage due to small-vessel occlusion, leading to nociceptive, neuropathic, and inflammatory pain [1]. Despite aggressive treatments, many patients with calciphylaxis experience severe pain, and limited literature exists into the optimal management of pain in this setting [1, 3]. 

Though calciphylaxis is a painful, debilitating, life-limiting disease, involvement of palliative care in patients with calciphylaxis is not well reported in existing literature and when present, is often late in the disease course [1, 3]. In the case of our patient, despite repeated and prolonged hospital admissions in the setting of multiple life-limiting prognoses and pain, it was only after a total of 6 months in hospital following the diagnosis of calciphylaxis that referral to palliative care was initiated. This possibly reflects lack of understanding of palliative care expertise in prescribing complex analgesic regimes and managing patient and caregiver needs [3]. A recently published clinical practice guideline in the United Kingdom by Chinnadurai et al. [1] supports referral to either a palliative medicine team and/or pain team when calciphylaxis is suspected due to its association with difficult-to-control pain and the additional morbidity associated with the use of inappropriate analgesia. 

Prescription of analgesics in patients with ESRF requires careful consideration of pharmacokinetics, most importantly the proportion of the drug and relevant active metabolites that are renally excreted, and whether there is removal of the drug during hemodialysis or peritoneal dialysis [4, 5]. Patients with ESRF are at increased risk of adverse drug effects, and dose reductions and avoidance of certain analgesics are required in patients with ESRF [4]. 

No uniform, evidenced-based strategy exists to support clinicians in the management of calciphylaxis-associated pain [1]. Chinnadurai et al.’s [1] clinical practice recommendations for the management of calciphylaxis-associated pain are extrapolated from literature on the management of pain in ESRF and expert opinion. Morphine, oxycodone, tramadol, and codeine should not be used for background analgesia due to the risk of opioid-induced neurotoxicity in this population; instead, transdermal fentanyl or buprenorphine is recommended [1]. Addressing the neuropathic element of pain secondary to calciphylaxis is important, and gabapentin or pregabalin are suggested; however, cautious dosing is required given gabapentinoids accumulate in patients with ESRF and are removed by dialysis [1]. Methadone can be considered if background pain remains problematic or dose-limiting toxicity is encountered [1]. However, there is very limited evidence in the literature of the efficacy of methadone for pain associated with calciphylaxis. To our knowledge, this is the first published case report of success of methadone as an adjunctive agent for pain in this setting, with one previous case report detailing analgesic treatment of calciphylaxis with levomethadone [6]. 

### Methadone use in palliative care 

Methadone is a synthetic opioid which also exerts activity at N-methyl-D-aspartate (NMDA), serotonin and norepinephrine receptors [7]. Its use as a second-line opioid for patients whose pain is poorly responsive to other opioids, or who develop dose-limiting adverse effects to conventional opioids, has increased significantly over the last two decades [8]. However, it has a complicated pharmacokinetic profile which limits its utility as a first-line analgesic option despite its low cost, long duration of action, oral bioavailability, observed safety in renal/hepatic insufficiency, and analgesic efficacy [7]. The activity of methadone as an NMDA receptor antagonist is considered to confer benefit in management of opioid-induced hyperalgesia, opioid tolerance, and (combined with serotonin and norepinephrine reuptake inhibition) neuropathic pain [8]. 

Methadone has a highly variable half-life amongst individuals, averaging 24 hours but can range from 12 hours to almost 1 week [8]. Consequently, the time to steady state could be anytime from several days to several weeks, and adjustment of doses should not occur more frequently than every 5 – 7 days [9]. In addition, methadone displays variable conversion ratios when rotating from other opioids. Consequently, prescription of methadone is usually restricted to clinicians experienced with its pharmacology and clinical application [8]. 

Methadone undergoes elimination via oxidative biotransformation involving multiple cytochrome P450 enzymes, and excretion into urine and feces [10]. There is no evidence of methadone accumulation in patients with ESRF [11, 12]. In patients with anuria, elimination of methadone and metabolites occurs almost exclusively via the fecal route [11]. In addition, methadone is virtually unremoved by peritoneal dialysis nor hemodialysis [11, 13]. Thus, it is appropriate to consider methadone for ESRF patients with complex analgesic requirements in consultation with a pain or palliative care specialist [12]. 

Cochrane reviews into methadone for cancer pain [14] and chronic non-cancer pain [15] are limited by very few randomized studies, and were unable to provide evidence of substantive efficacy of methadone in these settings. Methadone use in palliative care is thus largely guided by case reports/series and expert opinion [7]. Use of methadone in palliative care can involve either a complete rotation from a previous opioid to methadone, or as an adjunctive agent in lower doses [7, 8]. 

Methadone is associated with prolongation of the QT interval, torsades de pointes, and sudden cardiac death [15]. Baseline electrocardiogram assessment is preferable prior to commencement of methadone, with cautious use in patients with a QTc of > 450 ms and generally avoided if the QTc is > 500 ms [16]. We used a relatively low oral methadone dose at 2.5 mg daily for our patient given her QTc was approaching 450 ms. 

### Involvement of palliative care in patients with ESRF 

Palliative care has transitioned from primarily providing care to patients with cancer at the end of life, to a comprehensive service that focuses on symptom control, establishment of goals of care, psychosocial support, and coordination of care for patients with both malignant and non-malignant conditions [17]. However, patients with non-malignant conditions often have unpredictable clinical trajectories marked by exacerbations and periods of relative stability, making it difficult for patients and healthcare providers to determine when to transition to comfort-oriented approaches rather than focus on survival [18]. 

A recent Australian study found patients undergoing maintenance dialysis experience a median of nine symptoms, the most frequent being pain, shortness of breath, drowsiness, lack of energy, poor mobility, poor appetite, difficulty sleeping, pruritus, and depression/anxiety [19]. There is no doubt that patients with advanced kidney disease benefit from palliative care input [19]. However, involvement of palliative care in patients with ESRF in Australia varies by jurisdiction, and there are no national guidelines regarding appropriate referral pathways [20]. Whilst recognizing that patients with ESRF often have established and close relationships with their renal team, palliative care forms a valuable part of treatment and support for these patients whether they are undertaking dialysis or opting for a supportive non-dialysis pathway [20]. 

## Conclusion 

Palliative care often involves managing patients with poorly understood and under-researched conditions, such as calciphylaxis; using treatments with limited trial evidence, such as methadone; and consideration of altered pharmacokinetics in diverse clinical scenarios such as patients with renal failure. Early consultation with palliative care for patients with calciphylaxis is recommended to assist with complex pain management and holistic patient care. 

## Patient consent 

Informed consent was obtained from the patient. 

## Funding 

None. 

## Conflict of interest 

The authors declare no conflict of interest.
